# Antiulcer Activity of Anthraquinone–Flavonoid Complex of *Rumex tianschanicus* Losinsk

**DOI:** 10.3390/molecules28052347

**Published:** 2023-03-03

**Authors:** Gulnaz A. Seitimova, Aksholpan K. Shokan, Tatyana G. Tolstikova, Nataliya A. Zhukova, Dmitriy Yu. Korulkin, Nataliya O. Kudrina, Yuliya A. Litvinenko, Nataliya D. Meduntseva, Nina V. Terletskaya, Timur E. Kulmanov

**Affiliations:** 1 Faculty of Biology and Biotechnology and Faculty of Chemistry and Chemical Technology, Al-Farabi Kazakh National University, Al-Farabi Av., 71, Almaty 050040, Kazakhstan; 2 Institute of Genetic and Physiology, Al-Farabi Av., 93, Almaty 050040, Kazakhstan; 3 N.N. Vorozhtsov Novosibirsk Institute of Organic Chemistry, Siberian Branch of Russian Academy of Science, 630090 Novosibirsk, Russia

**Keywords:** *Rumex tianschanicus* Losinsk, anthraquinone, flavonoid, stomach ulcer, antiulcer effect

## Abstract

The composition of an ethanol extract from the roots of *Rumex tianschanicus* Losinsk of the Trans-Ili Alatau wild flora was studied in order to determine its antiulcer activity. The phytochemical composition of the anthraquinone–flavonoid complex from (AFC) *R. tianschanicus* revealed the presence of numerous polyphenolic compounds, the most abundant of which are anthraquinones (1.77%), flavonoids (6.95%), and tannins (13.39%). The use of column chromatography (CC) and thin-layer chromatography (TLC) in conjunction with UV, IR, NMR spectroscopy, and mass spectrometry data allowed the researchers to isolate and identify the major components of the anthraquinone–flavonoid complex’s polyphenol fraction: physcion, chrysophanol, emodin, isorhamnetin, quercetin, and myricetin. The gastroprotective effect of the polyphenolic fraction of the anthraquinone–flavonoid complex (AFC) of *R. tianschanicus* roots was examined in an experimental model of rat gastric ulcer induced by indomethacin. The preventive and therapeutic effect of the anthraquinone–flavonoid complex at a dose of 100 mg/kg was analyzed using intragastric administration per day for 1 to 10 days, followed by a histological examination of stomach tissues. It has been demonstrated that prophylactic and prolonged use of the AFC *R. tianschanicus* in laboratory animals resulted in significantly less pronounced hemodynamic and desquamative changes in the epithelium of gastric tissues. The acquired results thus offer fresh insight into the anthraquinone and flavonoid metabolite component composition of *R. tianschanicus* roots, and they imply that the examined extract can be used to develop herbal medicines with antiulcer activity.

## 1. Introduction

A stomach ulcer is formed as a result of a violation of the physiological balance between aggressive and protective factors of the mucous membrane. It has a rupture of the gastric mucosa, penetrating the muscularis mucosa and extending more than 5 mm in diameter [1]. When changes occur in the defense mechanisms of the stomach, it can cause changes in the gastric mucosa, which will eventually lead to erosion and then ulcers. Over the past 20 years, stomach ulcers have affected 5 to 10% of the world’s people and become a serious public health burden [2]. Long-term use of non-steroidal anti-inflammatory drugs (NSAIDs), alcohol consumption, tobacco smoking, pathogenic *Helicobacter pylori* infection (infectious agents), stress, prostaglandin levels, and antioxidant enzyme activity lead to peptic ulcer disease [3]. Damage to the gastric mucosa with the formation of an ulcer is the most common chronic disease of the gastrointestinal tract [4]. There is a link between chronic ulcers and stomach cancer. Gastric cancer is a complex multifactorial disease that proceeds in the form of successive events as a stepwise progression from inactive or chronic active gastritis to precancerous lesions of the stomach. For example, the prevalence of *H. pylori* infection can uniquely persist for decades in the harsh environment of the stomach, where it damages the gastric mucosa and alters the release pattern of gastric hormones, thereby affecting gastric physiology [1]. Exclusion of the consumption of aggressive nutritional components as well as the use of antisecretory, antacid, gastroprotective, and antibiotic medications are all necessary for the correction of the pathology associated with the development of defects in the gastric mucosa and the formation of an ulcer [5]. However, adverse effects related to a violation of the motor and digestive processes of the gastrointestinal system are typical of the majority of pharmacological drugs, particularly with their long-term use [6]. Significantly safer and no less effective in the treatment of diseases of the gastrointestinal tract can be the use of herbal medicine [7]. In this regard, it is acknowledged that the problem of developing low-toxic and highly effective herbal preparations for the treatment of pathologies associated with inflammation and damage to the gastric mucosa is recognized as relevant.

An important aspect of phytochemical research is the need to conduct parallel and cross-sectional studies, as well as the identification of new plant species with a high content of biologically active compounds. One of the most promising sources of antiulcer drugs is the Kazakh plants of the Polygonaceae Juss. The Kazakh species *R. tianschanicus* Losinsk is a perennial herbaceous plant belonging to the Polygonaceae family, widely distributed in Central Asia (Tien Shan) [8,9]. It is essential in pharmacology and widely used in folk medicine. *Rumex* roots were harvested in folk medicine at the end of the growing season (closer to autumn). They were used for skin diseases as an astringent. In veterinary medicine, water extracts of the fruits and roots of some species of *Rumex* were used as anti-inflammatory and wound-healing agents [10,11,12]. The presence of a variety of biologically active substances in the composition of various types of *Rumex*, as well as the associated therapeutic effect, necessitates further research on these plants. The polyphenol phytocomplex in numerous species of *Rumex* is mainly represented by anthraquinones, flavonoids and their glycosides, and tannins [13,14,15,16].

However, there have been few studies evaluating the effect of biologically active components derived from the species *R. tianschanicus*, and its antiulcer activity has not previously been evaluated. Therefore, the study of the antiulcer activity of the anthraquinone–flavonoid complex from (AFC) from a plant of the genus *Rumex* is of great practical interest.

The purpose of this research is to separate the AFC from the plant *R. tianschanicus*, identify the active principle, and determine the antiulcer activity of this complex in vivo.

## 2. Results

### 2.1. Identification of Major Components (1–6) of the Polyphenolic Fraction of R. tianschanicus Roots’ Anthraquinone–Flavonoid Complex

The assessment of the qualitative composition of the main groups of biologically active compounds was carried out on the basis of qualitative reactions. Phytochemical analysis of the AFC of *R. tianschanicus* proves the presence of many polyphenolic compounds in its composition, among which anthraquinones, flavonoids, and tannins dominate [17,18]. Quantitative determination of the content of main groups of biologically active compounds of AFC of *R. tianschanicus* roots was determined according to the methods of pharmacopoeial articles included in the collection of the State Pharmacopoeia of the USSR XI and the State Pharmacopoeia of Kazakhstan I-edition. Thus, in the AFC obtained from the roots of *R. tianschanicus*, the quantitative content of active substances was determined: anthraquinones—1.77%, flavonoids—6.95%, and tannins—13.39%.

**Figure 1:**
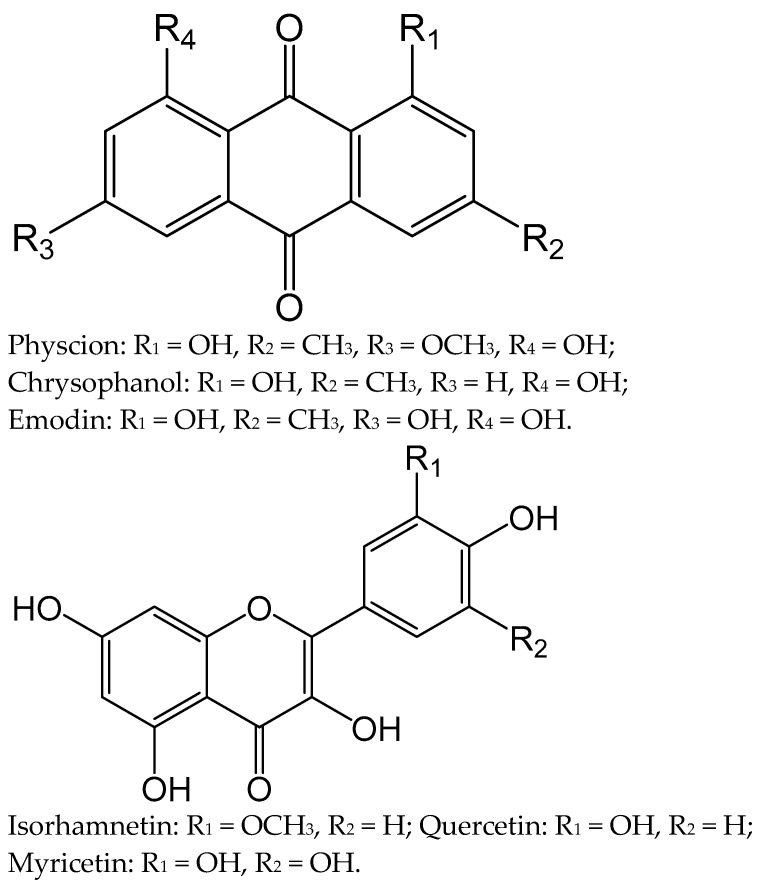
Structure of isolated compounds from *R. tianschanicus* roots.

Six known compounds were isolated from the AFC of *R. tianschanicus* (Figure 1), and their structures were elucidated through a combination of UV, IR, NMR, and MS spectroscopic analyses.

Compound **1**: Orange-yellow powder; C_16_H_12_O_5_; m.p. 201–202 °C; EI-MS, *m/z:* 284 [M]^+^; UV (MeOH) *λ_max_*: 221, 230, 252, 265, 428 nm; IR (KBr) *ν_max_*: 3400, 2970, 2825, 1680, 1628, 1490, 1370, 1320, 1275, 1220, 1160 cm^−1^; ^1^H NMR (400 MHz, CDCl_3_) *δ*: 12.53 (1H, s, OH-8), 12.07 (1H, s, OH-1), 7.80 (1H, br s, H-4), 7.35 (1H, s, H-5), 7.06 (1H, br s, H-2), 6.67 (1H, s, H-7), 3.93 (3H, s, -OCH_3_), 2.46 (3H, s, -CH3). ^13^C NMR (100 MHz, CDCl_3_) *δ*: 190.8 (C-9), 181.0 (C-10), 166.6 (C-6), 165.2 (C-8), 162.5 (C-1), 148.6 (C-3), 135.3 (C-11), 133.2 (C-14), 124.5 (C-2), 121.3 (C-4), 113.7 (C-13), 110.3 (C-12), 108.2 (C-5), 106.8 (C-7), 56.1 (OCH_3_), 22.2 (CH_3_). From these spectral data and comparison of previous reports [19,20,21], compound 1 was identified as physcion.

Compound **2**: Yellow powder; C_15_H_10_O_4_; m.p. 193–194 °C; EI-MS, *m/z:* 254 [M]^+^; UV (MeOH) *λ_max_*: 228, 258, 287, 429 nm; IR (KBr) *ν_max_*: 3400, 2960, 2890, 1670, 1628, 1570 cm^−1^; ^1^H NMR (400 MHz, CDCl_3_) *δ*: 12.14 (1H, s, OH-8), 12.00 (1H, s, OH-1), 7.98 (1H, d, J = 7.6 Hz, H-5), 7.70 (1H, dd, J = 7.8, 9.4 Hz, H-6), 7.55 (1H, s, H-4), 7.37 (1H, d, J = 7.8 Hz, H-7), 7.20 (1H, s, H-2), 2.45 (3H, s, -CH_3_). ^13^C NMR (100 MHz, CDCl_3_) *δ*: 192.5 (C-9), 181.9 (C-10), 162.7 (C-8), 162.4 (C-1), 149.3 (C-3), 136.9 (C-6), 133.6 (C-11), 133.2 (C-14), 124.5 (C-2), 124.3 (C-7), 121.4 (C-4), 119.9 (C-5), 115.8 (C-12), 113.7 (C-13), 22.3 (CH_3_). Compound 2 was identified as chrysophanol according to the compared spectral data [20,21,22,23].

Compound **3**: Orange needles; C_15_H_10_O_5_; m.p. 255–256 °C; EI-MS, *m/z:* 270 [M]^+^; UV (MeOH) *λ_max_*: 222, 253, 289, 438 nm; IR (KBr) *ν_max_*: 3400, 2970, 2850, 1670, 1635, 1595 cm^−1^; ^1^H NMR (400 MHz, DMSO-*d6*) *δ*: 12.07 (1H, s, OH-8), 12.01 (1H, s, OH-1), 11.40 (1H, s, OH-3), 7.55 (1H, s, H-4), 7.21 (1H, s, H-5), 6.85 (1H, s, H-2), 6.48 (1H, s, H-7), 2.38 (3H, s, -CH_3_). ^13^C NMR (100 MHz, DMSO-*d6*) *δ*: 189.4 (C-9), 180.9 (C-10), 165.4 (C-8), 164.3 (C-1), 161.2 (C-6), 148.0 (C-3), 134.8 (C-11), 132.5 (C-14), 123.9 (C-4), 120.2 (C-2), 113.2 (C-13), 108.7 (C-5), 108.6 (C-12), 107.7 (C-7), 21.4 (CH_3_). Compound 3 was recognized as emodin according to the compared spectroscopic data [19,24,25].

Compound **4**: Yellow crystals; C_16_H_12_O_7_; m.p. 303–304 °C; EI-MS, *m/z:* 316 [M]^+^; UV (MeOH) *λ_max_*: 370, 278, 254 nm; IR (KBr) *ν_max_*: 3450, 2980, 1680, 1570, 1515 cm^−1^; ^1^H NMR (400 MHz, CD_3_OD) *δ*: 7.85 (1H, s, H-2′), 7.51 (1H, d, J = 8.0 Hz, H-6′), 6.88 (1H, d, J = 8.3 Hz, H-5′), 6.33 (1H, s, H-8), 6.18 (1H, s, H-6), 3.89 (3H, s, OCH_3_). ^13^C NMR (100 MHz, CD_3_OD) *δ*: 177.4 (C-4), 165.6 (C-7), 160.4 (C-5), 157.5 (C-9), 156.8 (C-2), 148.8 (C-3ʹ), 147.9 (C-4ʹ), 138.4 (C-3), 124.5 (C-1′), 121.4 (C-6′), 116.1 (C-5′), 115.4 (C-2′), 104.5 (C-10), 99.3 (C-6), 94.4 (C-8). After a comparison of these data with the reported studies, compound 4 was confirmed to be isorhamnetin [26].

Compound **5**: Yellow crystals; C_15_H_10_O_7_; m.p. 310–311 °C; EI-MS, *m/z:* 302 [M]^+^; UV (MeOH) *λ_max_*: 372, 268, 256 nm; IR (KBr) *ν_max_*: 3402-3120, 1665, 1612, 1560, 1518, 1449, 1382, 1318, 1262, 1168, 1014 cm^−1^; ^1^H NMR (400 MHz, DMSO-*d6*) *δ*: 8.62 (1H, s, H-2′), 7.86 (1H, d, J = 8.5 Hz, H-6′), 7.35 (1H, d, J = 8.5 Hz, H-5′), 6.72 (1H, s, H-8), 6.76 (1H, s, H-6). ^13^C NMR (100 MHz, DMSO-*d6*) *δ*: 177.3 (C-4), 165.7 (C-7), 162.5 (C-5), 158.2 (C-9), 147.9 (C-2), 148.7 (C-4′), 146.0 (C-3′), 137.2 (C-3), 124.1 (C-1′), 121.6 (C-6′), 116.2 (C-5ʹ), 116.0 (C-2ʹ), 104.4 (C-10), 99.3 (C-6), 94.4 (C-8). On the basis of spectral data and in comparison with literature sources, the structure of compound 5 was determined and identified as quercetin [27].

Compound **6**: C_15_H_10_O_8_, yellow-green crystalline powder, m.p. 364–365 °C. EI-MS, *m/z*: 318 [M]^+^. UV (MeOH) *λ_max_*: 374, 258 nm; IR (KBr) *ν_max_*: 3385–3300, 1660, 1565, 1516 cm^−1^; ^1^H-NMR (400 MHz, CD_3_OD) *δ*: 7.34 (2H, s, H-2′, H-6′), 6.37 (1H, s, H-8), 6.17 (1H, s, H-6). ^13^C NMR (100 MHz, CD_3_OD) *δ*: 176.3 (C-4), 164.3 (C-7), 161.2 (C-5), 156.1 (C-9), 155.5 (C-2), 146.5 (C-3′), 136.5 (C-3), 134.4 (C-4′), 120.5 (C-1′), 108.1 (C-2′, C-6′), 102.2 (C-10), 98.6 (C-6), 93.3 (C-8). The structure of compound 6 was established as myricetin after comparing it with the spectral data from the previous studies [27,28].

### 2.2. Effect of Anthraquinone–Flavonoid Complex from R. tianschanicus on Body Weight and Organ Mass Ratios in Rats in an Indomethacin Gastric Ulcer Experimental Model

Table 1 shows the results of measuring body weight and organ mass ratios in healthy rats from the control group and the group with induced indomethacin ulcers who were given AFC of *R. tianschanicus* orally.

As follows from the presented data, the body weight of experimental animals in the group with gastric ulcer was significantly lower than in the control group (*p* < 0.05) and groups (Prophylactic injection, effect) with the introduction of an AFC based on *R. tianschanicus* was significantly (*p* < 0.05) higher than in the control group of rats. In the studied organs, such as the liver and stomach, the group with gastric ulcers had significantly lower levels than the control group (*p* < 0.05), while the Prophylactic injection group had significantly higher levels (*p* < 0.05) than in the control group rats.

### 2.3. Effect of Anthraquinone–Flavonoid Complex R. tianschanicus on Models of Indomethacin Damage to the Gastric Mucosa in Rats

The results of the antiulcer activity of the AFC of *R. tianschanicus* in models of indomethacin damage to the gastric mucosa in rats are presented in Table 2.

The study’s results demonstrated that a single intragastric administration of the AFC of *R. tianschanicus* at a dose of 100 mg/kg had no antiulcer activity, whereas prolonged use and prophylactic administration had a positive effect on the dose used. The effectiveness of the AFC of *R. tianschanicus* was characterized by a decrease not only in large, point, strip-like ulcers but also in the number of ulcerative lesions in rats in the group with a prophylactic injection of parenteral nutrition was Section 2.3, and prolonged use was Section 2.1.

### 2.4. Pathological Study of Experimental Animals’ Stomachs

**Figure 2:**
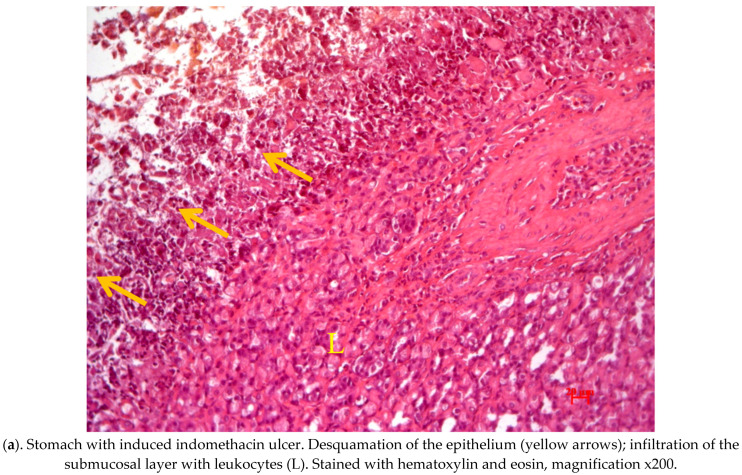

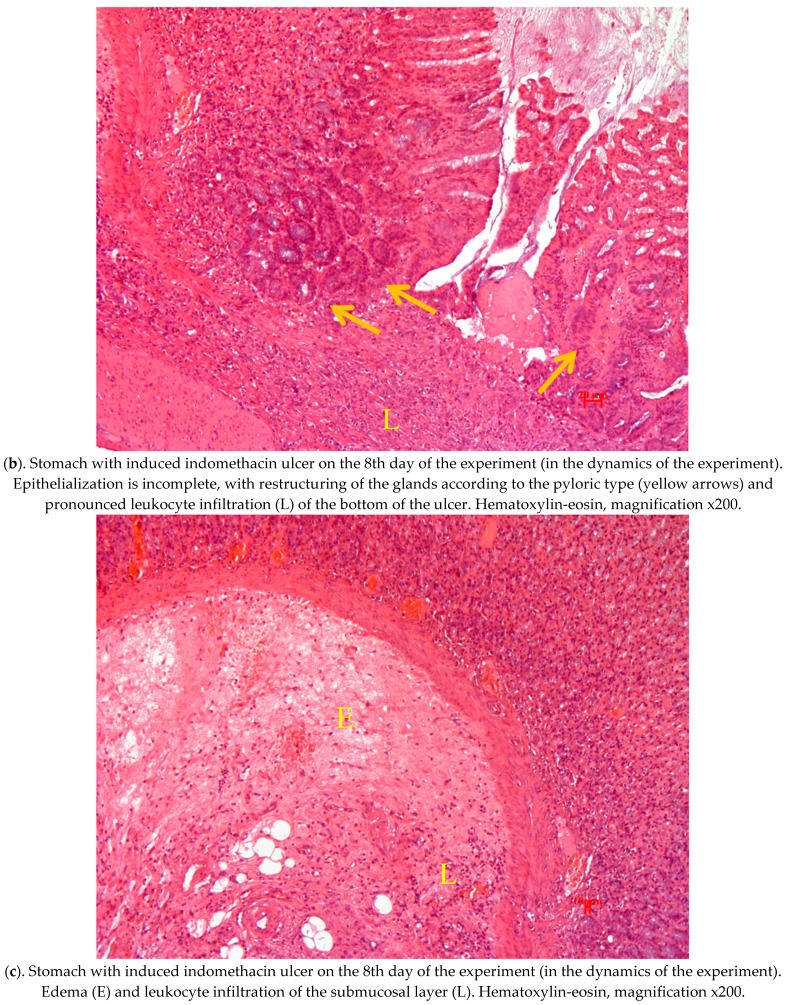

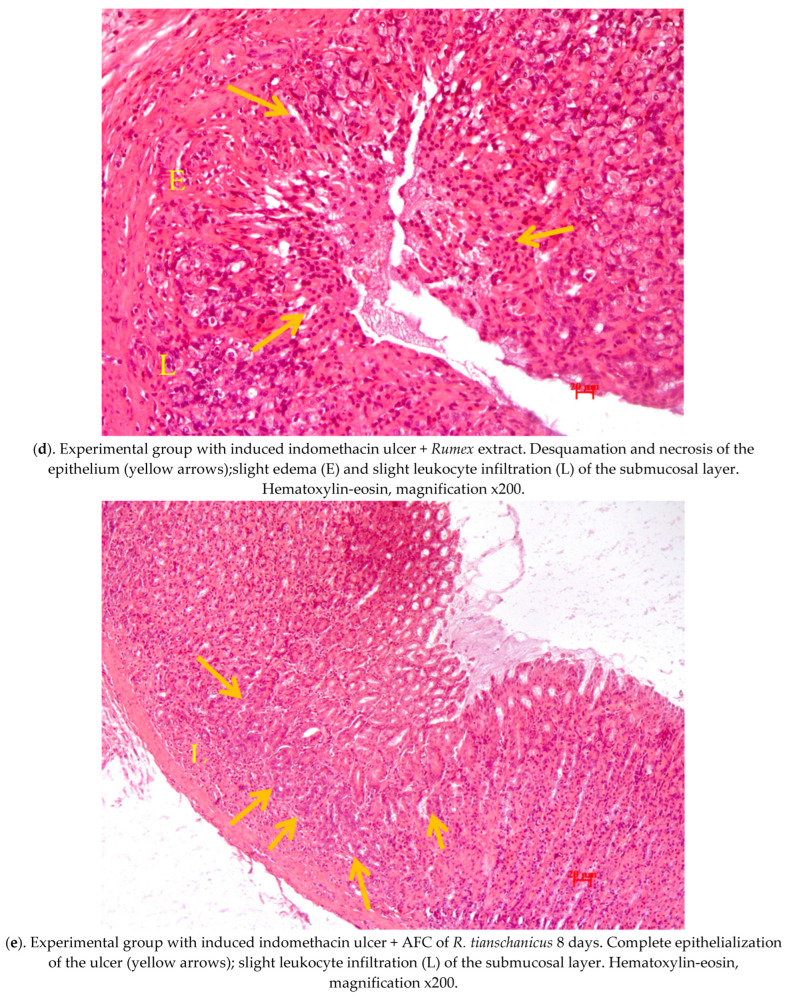

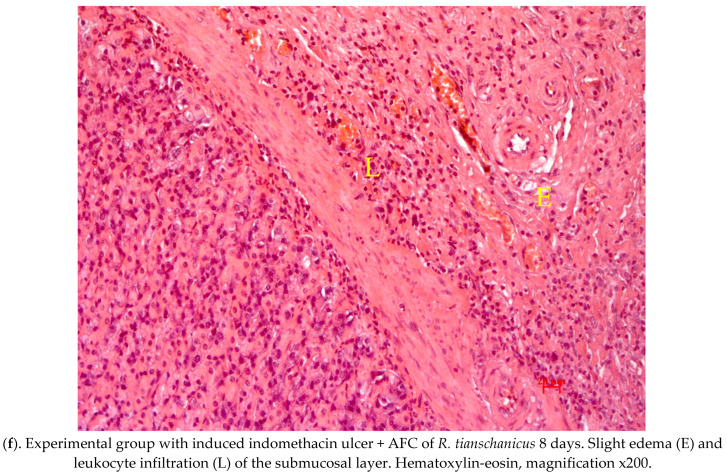
(**a**) desquamation of the epithelium, infiltration of the submucosal layer with leukocytes; (**b**) incomplete epithelialization, with restructuring of the glands according to the pyloric type and pronounced leukocyte infiltration of the bottom of the ulcer; (**c**)edema and leukocyte infiltration of the submucosal layer; (**d**) desquamation and necrosis of the epithelium and slight edema and slight leukocyte infiltration of the submucosal layer; (**e**) complete epithelialization of the ulcer and slight leukocyte infiltration of the submucosal layer; and (**f**) slight edema and leukocyte infiltration of the submucosal layer.

Figure 2 shows the results of a pathomorphological study of the stomach of rats of the control and experimental groups with indomethacin-induced ulcers, which were orally administered AFC of *R. tianschanicus.*

Figure 2 also shows the histological sections of the stomachs of rats from different groups of animals and changes in the stomach of rats with indomethacin ulcer and its treatment AFC of *R. tianschanicus* Hematoxylin-eosin, magnification x200.

The stomach wall was represented in laboratory animals (rats) by a glandular section located in the central part of the stomach along the greater curvature. The cardiac and partial bordering with the pyloric parts of the department were noted in the preparations, with different heights of the glandular epithelium.

The results of histological studies in the control group of animals treated with indomethacin at a dose of 25 mg/kg for ulcer induction revealed a pronounced infiltration of polymorphonuclear leukocytes of the own plastics of the mucosa and submucosa in the cardiac part of the stomach at all stages of the experiment (Figure 2a). Mast cells and macrophages are often found in the submucosa. In the experimental gastric ulcer group, the capillaries and vessels are plethoric, and diapedesis hemorrhages are observed in the submucosal and muscular layers. The glandular epithelium is edematous, with pronounced desquamative changes that are focal in nature. Deeper defects in the mucous membrane were also found: focal epithelium desquamation reached half the thickness of the mucous membrane, and in some animals, the epithelium defect extended to the entire thickness (Figure 2b). In the prophylactic administration group, partial epithelialization of ulcerative defects was observed, and inflammatory cell infiltration slightly decreased while maintaining venous plethora (Figure 2c). In the group of animals that received a single injection, the ulcer epithelized with an incomplete mucosal defect from the bottom of the glands, with complete restoration of the defect. Whereas with the epithelium lesion through the entire thickness to the submucosa, the epithelization was incomplete, with signs of pyloric-type gland restructuring beginning from the edges of ulcerative defects (Figure 2d,e).

Less pronounced hemodynamic and desquamative changes in the epithelium were observed in animals with long-term use throughout the experiment (Figure 2f). Mucosal defects were superficial, and epithelialization was complete but not accompanied by pyloric epithelial restructuring.

## 3. Discussion

A chemotaxonomic analysis of the distribution of natural compounds in Polygonaceae individual phyla can be the basis for predicting the search for valuable biologically active substances [29,30].

The prerequisites for this work were ethnopharmacological studies of plants of the genus *Rumex* in functional disorders of the gastrointestinal tract and gastritis [11,31,32]. It has been shown that plants of the genus *Rumex* L. are rich in secondary metabolites of the polyphenol type, and biologically active components isolated from medicinal plants exhibit antiulcer activity through different mechanisms of action [11,15,16,31,32]. It is known that extracts from the roots of some *Rumex* species were used in homeopathy, in Chinese and Indian medicine as an astringent, laxative, antidysenteric, and stomach tonic, used for various gastrointestinal diseases, intestinal ulcers, enterocolitis, and gastritis [12,31,32,33]. For example, horse sorrel (*R. confertus* Willd.) is used as a symptomatic remedy in the treatment of malignant tumors and stomach ulcers [11]. Sisay Z.W. et al. reported that crude methanol extract and diluent fractions of *R. nepalensis* root showed promising antiulcer activity in pylorus ligation [34]. Ji-Yeong Bae et al. have shown that an extract derived from *Rumex acetosa* has antiulcerogenic activity in mice [35].

Analysis of the literature data shows that out of 23 species of *Rumex* in the flora of Kazakhstan, about 20 species have been studied chemically. The phytochemical composition of the AFC obtained by us based on *R. tianschanicus* was evaluated, and the analysis confirmed the presence in the composition of polyphenolic compounds characteristic of the Polygonaceae families with a predominance of anthraquinones, flavonoids, and tannins [17,18]. As a result of this study, six known compounds of flavonoid (isorhamnetin, quercetin, and myricetin) and anthraquinone (physcion, chrysophanol, and emodin) nature were isolated from the AFC of *R. tianschanicus.* As shown in other studies, these compounds have anti-inflammatory and antiulcer activity and can be used in the treatment of gastrointestinal diseases [18,19,36,37,38,39,40,41]. Indomethacin (NSAIDs) is known to cause damage to the gastric mucosa by activating inflammatory cells, producing anti-inflammatory cytokines, and inducing oxidative stress [42,43]. In our experiment, indomethacin induced the gastric ulcer because it is a convenient experimental model and effectively causes damage to the gastric mucosa.

As shown by Sisay Z.W. et al., the resulting complex of secondary metabolites, namely flavonoids in the closely related species of *R. tianschanicus*, *R. nepalnesis* can stimulate important cellular mechanisms, such as proliferation and migration of epithelial cells, has a cytoprotective effect, and triggers the release of prostaglandins [42]. Elsanhoty R.M. et al. also demonstrated that anthraquinones stabilize mast cells, assisting in the maintenance of optimal microcirculation in damaged tissues, reducing histamine production, and weakening the damaging effect of the ulcerogen [44]. Our results fully correspond with and confirm the data of other researchers presented earlier, and taking into account the obtained antiulcer effect of anthraquinones and flavonoids, it is logical to assume that the gastroprotective function of *R. tianschnicus* is based on the complex local effect of the AFC on the damaged area of the gastric mucosa. This thesis is also confirmed by a macromorphological assessment of the surface of the gastric mucosa (GM) and counting the number of affected areas, which we expressed using the Pauls’ index [45] against the background of prophylactic and prolonged intragastric management of the AFC of *R. tianschanicus*. Therefore, the complex’s antiulcer activity index was 2.1 for long-term use and 2.3 for prophylactic use. If the result is greater than 2.0, as demonstrated by the authors, we are referring to positive antiulcer activity.

The complex restored the gastric membranes’ protective ability against the aggressive effects of indomethacin, with a concomitant increase in prostaglandin synthesis, postulating its role in increasing the amount of mucus and its inclusion in ulcer prevention [46,47,48,49].

As a result, it can be concluded that the AFC based on *R. tianschanicus* may have a much broader spectrum of physiological action, in addition to the identified antiulcer activity, and is thus promising for further phytochemical and pharmacological research.

## 4. Materials and Methods

### 4.1. Plant Material Collection and Preparation

*R. tianschanicus* plant materials were harvested in June 2021 in the foothills of the Zailiyskiy Alatau (Almaty region, South-Eastern Kazakhstan) at the coordinates N 43.1585, E 77.2688, at an altitude of 2250 m above sea level. The underground part is made up of many-headed rhizomes, and the root is thick, slightly branched, and taproot-like. The certificate of identification of harvested plants (N 01-06/144) was issued by specialists from the Institute of Botany and Phytointroduction of the Ministry of Ecology, Geology and Natural Resources of the Republic of Kazakhstan. *R. tianschanicus* underground organs were extracted using the air-shadow method.

### 4.2. General Experimental Procedures

UV spectra were recorded on a Shimadzu UV-1601PC spectrophotometer in spectroscopic grade MeOH. IR spectra were recorded on a Shimadzu IR-460 spectrometer in pressed KBr disks. NMR spectra were recorded on Bruker AMX 400, operating at 400 MHz for proton and 100 MHz for carbon in spectroscopic grade CDCl_3_, DMSO-*d6*, and CD_3_OD. EI-MS (70 eV) were recorded on Agilent 7890A/5975C. TLC was carried out on precoated silica gel 60 F 254 aluminum sheets (Merck, Rahway, NJ, USA). Whatman chromatography paper (Grade 1 CHR) was obtained from GE Healthcare Life Sciences (Shanghai, China) and used with further adjustment of size. All chromatographic solvents were of reagent grade (Sinopharm Chemical Reagent Co., Ltd., Shanghai, China). For column chromatography, normal phase silica gel 60 (70–230 mesh, Merck), Sephadex LH-20 (25–100 μm, Sigma-Aldrich, St. Louis, MO, USA), and polyamide (particle size 50–160 μm, Sigma-Aldrich) were used.

### 4.3. Extraction and Isolation

The air-dried roots (1 kg) were exhaustively extracted with 50% ethanol for 72 h at room temperature, twice. The extracts were combined and concentrated into a thick extract under reduced pressure.

An equal volume of a saturated solution of zirconium nitrate was added to the obtained extract to obtain a polyphenol (anthraquinone–flavonoid) fraction, and the formed precipitate was separated by centrifugation in a CN-650 HT Machinery centrifuge (5000 rpm). The zirconium was removed from the precipitate by adding a 3% solution of sodium oxalate until the solution became translucent. The zirconium oxalate precipitate was separated by centrifugation.

Quantitative determination of anthraquinones was carried out according to the pharmacopoeia procedure, CoCl_2_·6H_2_O was used as a standard for constructing a calibration curve at 525 nm; flavonoids were estimated by differential spectrophotometry with aluminum chloride at an analytical wavelength of 430 nm, expressed as quercetin; tannin contents were determined using a pharmacopoeia method (permanganometric method) [50,51,52].

For complete separation of the polyphenol complex, sequential extraction of created fraction of 50% ethanol extract with benzene and ethyl acetate was carried out. Each extract was separately evaporated to dryness using rotary vacuum evaporator under reduced pressure at a temperature not exceeding 45 °C. The resulting extracts were (23 g and 47 g, respectively). Each of the obtained fractions was subjected to thin layer (TLC) and paper (PC) chromatographic techniques and then column chromatography (CC) for isolation of its major constituents.

The benzene fraction obtained above was fractionated on a silica gel column using benzene–methanol. Elution was begun with benzene, and polarity was gradually increased in a gradient elution technique and finally ended with methanol. The elute was collected in fractions, each fraction was concentrated under reduced pressure and monitored by silica gel plates. Based on thin layer chromatography (TLC) profiles, the fractions were combined to give four fractions A–D (A: 1–20; B: 21–30; C: 31–45; D: 46–55). Final purification of fraction B was carried out on preparative TLC plates using petroleum ether–methanol solvent systems to isolate compounds **1** (31 mg) and **2** (29 mg). Fraction C was purified on Sephadex LH-20 gel column using MeOH as eluent to give compound **3** (35 mg). Fraction D underwent column chromatography on silica gel with the petroleum ether–methanol system to yield compound **4** (32 mg).

The ethyl acetate extract was subjected to polyamide column chromatography. The column was successively eluted with different ratios of water and ethanol. Fractions of 50 mL each were collected, and similar fractions were pooled together after screening by TLC to four main fractions A–D (A: 1–5; B: 6–13; C: 14–32; D: 33–42). Fraction C was rechromatographed over silica gel using chloroform–ethanol (from 9:1 to 1:1) as eluent to yield compounds **5** (41 mg) and **6** (28 mg).

The isolated compounds (**1**–**6**) were characterized by comparing their spectroscopic data (UV, IR, ^1^H NMR, ^13^C NMR, and MS) with those previously published in the literature.

### 4.4. Animals

All preclinical animal experiments were conducted with the approval of the ethical commission “Institute of Human and Animal Physiology” of the CS MES RK, which was valid from 1 January 2021 to 31 December 2022 under Protocol No. 07-05/68 dated 17 June 2020. White laboratory Wistar rats weighing 220–250 g were fed a vivarium diet under standard conditions. The animals were weighted randomly and divided into groups of six. As bedding, sawdust was used. The air temperature in the vivarium rooms was kept between 19 and 21 °C, with a relative humidity of 55 to 65%. The animals received proper care daily. The animals were obtained from the vivarium of the Institute of Cytology and Genetics of the Siberian Branch of the Russian Academy of Sciences and were kept in special vivarium installations in IVC systems of individually ventilated cages SK-MVCS-70RRD (South Korea) with free access to food and water. All manipulations with animals were carried out in strict accordance with the Order of the Ministry of Health of the Russian Federation No. 199n “Rules for Good Laboratory Practice” dated 1 April 2016 and the provisions of Directive 2010/63/EU of the European Parliament and the Council of the European Union dated 22 September 2010 on the protection of animals used for scientific purposes, as well as the requirements and recommendations of the Guidelines for the Care and Use of Laboratory Animals. The study was conducted under the supervision of Professor T.G. Tolstikova in the laboratory of pharmacological research of the N.N. Vorozhtsov Novosibirsk Institute of Organic Chemistry, Siberian Branch of Russian Academy of Science, Russian Federation, Al-Farabi KazNU of the Republic of Kazakhstan, and the Institute of Genetics and Physiology of the Republic of Kazakhstan.

### 4.5. Experiment Design

Indomethacin gastric ulcer was modeled on 36 female rats according to the standard method [43].

Group 1. Control group—water-tween mixture was administered intragastrically.

Group 2. Experimental group with gastric ulcer induced by indomethacin without treatment. Animals were injected with indomethacin substance (Tokyo Chemical Industry Co., Ltd., Tokyo, Japan) at a dose of 25 mg/kg per os.

Group 3. Prophylactic administration of AFS of *R. tianschanicus* at a dose of 100 mg/kg.

Group 4. Single injection of AFC of *R. tianschanicus* at a dose of 100 mg/kg. An hour later, the animals were injected with the substance indomethacin (Tokyo Chemical Industry Co., Ltd.) at a dose of 25 mg/kg per os.

Group 5. Administration of AFC of *R. tianschanicus* at a dose of 100 mg/kg for 10 days (long-term effect). An hour later, the animals were injected with the substance indomethacin (Tokyo Chemical Industry Co., Ltd.) at a dose of 25 mg/kg per os.

The animals were euthanized with CO_2_ after the experiment was completed. The stomachs were removed and opened along the lesser curvature to assess the general condition of the gastric mucosa and count the number of ulcerative defects. The presence of macroscopic changes in the structure of the stomach, liver, kidneys, heart, and pancreas was also evaluated. The material was then fixed in 10% neutral formalin. According to the results of examination of the stomachs of experimental animals, the index of antiulcer activity (AA) was calculated for each test compound.

The Pauls’ index (*PI*) was calculated for each experimental group using the following formula:

$$PI=\frac{A\times B}{100},$$

where *A* is the average number of ulcerative defects per animal and *B* is the percentage of animals in the group with ulcers.

The antiulcer activity index was calculated as the ratio of the Pauls’ index for the control group to the index of the experimental group:

$$AA=\frac{PI_{control}}{PI_{experiment}}$$

Antiulcerogenic activity was assigned to substances with an *AA* index greater than or equal to 2 [46].

All animal studies were conducted in accordance with ethical principles as well as the guidelines “Rules for conducting preclinical studies, biomedical experiments, and clinical trials in the Republic of Kazakhstan” (dated 25 July 2007, No. 442).

### 4.6. Histological Examinations

Rat stomachs were fixed in 10% neutral formalin for histological examination, followed by standard processing on the MICROM histological complex and embedding in paraffin blocks. Hematoxylin and eosin were used to stain sections 3–4 µm thick. The degree of stomach damage was determined using the following criteria: the presence of desquamative changes, mucosal defects of varying depths, and the degree of infiltrative changes [53].

### 4.7. Statistical Data Processing

The Student’s t-test was used at *p* ≤ 0.05 to compare different values between samples (Statistica 7, StatSoftInc., St. Tulsa, OK, USA). The obtained morphometric data were processed using MS Excel’s capabilities. The standard error of the sample mean was calculated, as was the standard error. The t-test of significance was used to calculate the significance of the difference between the indicators of the control and experimental groups, the *p* value was obtained from the Student’s value table, and changes were considered significant at *p* ≤ 0.05. All data is computed.

## 5. Conclusions

For the first time, we examined the impact of *R. tianschanicus* anthraquinone–flavonoid complex on indomethacin-induced gastric ulcers in rats. Based on the analysis of the data on morphological parameters and effectiveness of the treatment, indomethacin-induced gastric ulcer experimental therapy with an AFC derived from the roots of *Rumex tianschanicus* Losinsk was found to be effective in treating and restoring the epithelium of the stomach wall.

The revealed antiulcer activity, as well as the presence of secondary metabolites with potential therapeutic activity, makes *Rumex tianschanicus* Losinsk a promising medicinal raw material for the development of phytopreparations based on it.

## Figures and Tables

**Table 1:** Body mass indices and organ mass coefficients in rats in the absence of indomethacin gastric ulcer induction and in the presence of AFC of *R. tianschanicus* treatment.

Animal Groups	Total Body Weight, g	Heart, g	Kidneys, g	Liver, g	Stomach, g	Spleen, g
Control	225.5 ± 7.2	1.8 ± 0.4	1.7 ± 0.5	8.9 ± 0.6	9.6 ± 0.6	3.6 ± 0.4
Stomach ulcer	210.2 ± 8.2 *	1.6 ± 0.3	1.8 ± 0.4	7.8 ± 0.5 *	8.3 ± 0.5 *	3.3 ± 0.4
Prophylactic injection	238.4 ± 6.7*	1.9 ± 0.6	1.9 ± 0.4	9.2 ± 0.6	10.2 ± 0.9 *	3.9 ± 0.6
Single injection	228.4 ± 5.9	2.1 ± 0.6	1.8 ± 0.5	9.1 ± 0.7	9.8 ± 0.8	3.7 ± 0.7
Long-lasting effects	255.5 ± 9.2 *	2.2 ± 0.2	2.1 ± 0.5	10.1 ± 0.5 *	10.7 ± 0.7 *	4.1 ± 0.6

Note: *—statistically significant changes in the control group in relation to the other group, at *p* ≤ 0.05.

**Table 2:** Antiulcer activity of intragastric administration of AFC of *R. tianschanicus* in models of indomethacin damage to the gastric mucosa in rats.

Animal Groups	Rat Ulcer Prevalence, %	Number of Punctate Ulcers	Number of Large Ulcers	PI	AA
Control	100	4.8 ± 0.9	4.6 ± 0.7	12.3	-
Stomach ulcer	92	3.5 ± 0.4	4.1 ± 0.6	7.4	1.5
Prophylactic injection + Stomach ulcer	60	2.1 ± 0.4 *	1.2 ± 0.5 *	6.2	2.3
Single injection + Stomach ulcer	35	2.7 ± 0.6 *	2.8 ± 0.8 *	5.4	1.9
Prolonged use + Stomach ulcer	30	1.3 ± 0.5 *	1.8 ± 0.3 *	6.1	2.1

Note: *—statistically significant changes in the control group in relation to the other group, at *p* ≤ 0.05; PI—Pauls’ index; AA—antiulcer activity.

## Data Availability

Not applicable.
